# Non-celiac gluten sensitivity as a rare cause of growth retardation in children: a case series study 

**Published:** 2021

**Authors:** Hedyeh Saneifard, Ali Sheikhy, Fereshteh Karbasian, Golnaz Eslamian, Marjan Shakiba, Delara Babaie

**Affiliations:** 1 *Department of Pediatric Endocrinology and Metabolism, Mofid Children Hospital, Shahid Beheshti University of Medical Sciences, Tehran, Iran*; 2 *Research Department, Tehran Heart Center, Tehran University of Medical Sciences, Tehran, Iran*; 3 *Department of Pediatric Emergency, Mofid Children Hospital, Shahid Beheshti University of Medical Sciences, Tehran, Iran*; 4 *Department of Allergy and Clinical Immunology, Mofid Children Hospital, Shahid Beheshti University of Medical Sciences, Tehran, Iran*

**Keywords:** Non-celiac gluten sensitivity, Gluten-free diet, Failure to thrive, Growth

## Abstract

**Aim:**

Herein, we present five children and adolescents with a final diagnosis of non-celiac gluten sensitivity (NCGS).

**Background:**

Non-celiac gluten sensitivity (NCGS) is a condition characterized by gastrointestinal and extra-intestinal symptoms triggered by ingestion of gluten-containing compounds, e.g., wheat, rye, and barley, in subjects without celiac disease or wheat allergy.

**Methods:**

Demographic characteristics, clinical manifestations, serum biomarkers and skin prick test were evaluated. Patient data was also recorded after they followed a gluten-free diet (GFD). Height and weight were measured, and all patients were examined 6 months after following the suggested GFD.

**Results:**

All patients had failure to thrive and abdominal pain. Clinical symptoms were reduced, and significant weight and height gains were detected after 1 month of following a gluten-free diet.

**Conclusion:**

The relationship between failure to thrive (FTT) and NCGS is still unknown; hence, NCGS may be one of the main causes of FTT which can be prevented by gluten-free diets.

## Introduction

 Non-celiac gluten sensitivity (NCGS) causes intestinal and extra-intestinal symptoms triggered by the introduction of gluten-containing foods (e.g., wheat, rye, and barley) in the absence of celiac-specific antibodies and villous atrophy as well as of any allergy related processes (1, 2). Symptoms can often resemble celiac disease. Abdominal pain, bloating, and altered bowel habits suggesting gastrointestinal symptoms and fatigue, headache, bone or joint pain, mood disorders, and skin manifestations (e.g., eczema or rash) are systemic symptoms (3-5). NCGS may be diagnosed only after the exclusion of celiac disease (CD), and wheat allergy as an established serological marker is not yet available (6).

The overall prevalence of NCGS in the general population is still unknown, mainly because many patients are currently self-diagnosed and start a gluten-free diet (GFD) without medical advice or consultation. The approximate prevalence rate of NCGS ranges between0.5% and 13% in the general population based on self-reported data (7-10). In addition, prevalence is higher in women (7, 8), teenagers, and patients in the third decade of life (7, 9).

The concept of NCGS was described for the first time in 1978 by Ellis et al. (11). Their article described some patients with celiac symptoms such as abdominal pain and diarrhea with no histological duodenal lesions who improved following a GFD. In 1980, Cooper et al. (12) reported 8 females with abdominal pain, diarrhea, and normal duodenal histology who improved with a GFD, but experienced a relapse of symptoms following a gluten challenge. The pathophysiology of NCGS is largely unknown. To date, three main hypotheses have been suggested to cause symptoms. The first one is gluten, which represents the major storage proteins of wheat, barley, and rye, in the endosperm of grains. The second factor is fermentable oligosaccharides, disaccharides, monosaccharides, and polyols (FODMAPs) that may play a significant role in NCGS pathophysiology and symptom development. The last predisposing factor is amylase-trypsin inhibitors, which represent up to 4% of total amount of grain proteins (13). However, 25%to 56% of subjects with NCGS had positive (>50 arbitrary units) anti-gliadin IgG (4, 14). Herein, we present a case series of 5 patients with FTT who were diagnosed with NCGS. 

## Methods

**Table 1:** Symptoms and growth indices data before and after 6 months of GFD in patients with NCGS^*^

Patients	Age (years)	Sex	Height	Weight	Symptoms	Duration of symptoms	Height gain (Final percentile)	Weight gain (Final percentile)
No. 1	4.5	Male	100 cm (10^th^ percentile)	14 Kg (3^rd^ percentile)	Abdominal pain/ constipation/ acute urticaria	Within the previous year	4 cm (25^th^ percentile)	2.7 Kg (25^th^ percentile)
No. 2	16.89	Male	130 cm (Z score= -5.55)	26.7 Kg (Z score= -8.21)	Abdominal pain/ nausea/ IBS like symptoms	6 years	2 cm (Z score= -5.4)	3.6 Kg (Z score= -6.8)
No. 3	3.5	Female	97.5 cm (50^th^ percentile)	12.9 kg (10^th^ percentile)	Abdominal pain/ asthma/ eczema/ urticaria	New onset	3 cm (50^th ^percentile)	1.5 Kg 25^th^ percentile)
No. 4	8.5	Female	117.5 cm (Z score= -2.27)	21.5 Kg (5^th^ percentile)	Abdominal pain	New onset	4 cm (Z score= -1.94)	2 Kg (10^th^ percentile)
No. 5	6.66	Female	106 cm (Z score= -2.6)	17.2 Kg (3^rd^ percentile)	Abdominal pain/ constipation	New onset	3 cm (Z score= -2.46)	2.4 Kg (10^th^ percentile)

* GFD: Gluten free diet; NCGS: Non-celiac gluten sensitivity

All pediatric cases were diagnosed in Mofid Children’s Hospital (Tehran, Iran) between Feb. 2018 and Feb.2019. Five cases were identified as having a “non-celiac gluten sensitivity” diagnosis. This study included patient’s demographic characteristics, clinical manifestations, serum biomarkers (e.g., anti-tTG), and skin prick test results. The samples for IgA anti-tTG were analyzed using ELISA kits based on solid-phase enzyme immunoassays (Kit: CeliAK IgA LINE-4208, Generic Assay, Berlin, Germany). Patient data was also recorded after they followed a GFD. Employing a standard stadiometer, height was measured with subjects standing; the nearest 0.1 cm was recorded. Weight was assessed with a digital scale (Beurer, GS49, Germany), and only light clothing was allowed. All patients were examined6 months after following the suggested GFD. This study was approved by the ethics committee of Shahid Beheshti University of Medical Science. 

## Results

**Case 1**

A 4.5-year-old boy referred by a pediatric endocrinologist presented with abdominal pain, short stature, and low weight. His growth indices at his first visit were height for age: 10^th^ percentile and weight for age: 3^rd^ percentile. Despite a normal height percentile, the patient was shorter by more than two standard deviations from his mid-parental height. Following the mentioned symptoms, anti-trans glutaminase antibody and skin prick tests were performed to evaluate for CD and wheat sensitivity, respectively. Both tests were negative, and the patient had no IgA deficiency. A GFD was suggested for the patient with suspicion of NCGS. The patient’s abdominal pain was relieved within one month of starting the GFD, and his growth indices improved as follows: height from 100 cm to 104 cm and weight from 14 Kg to 16.7 Kg.

**Case 2**

A 17-year-old boy was referred by a pediatric gastroenterologist with a history of abdominal pain and nausea. He was a severe case of FTT with a bone age of 8 years and weight of8.5 years. He had been under treatment for suspicion of irritable bowel syndrome (IBS) for almost 6 years, but there was no improvement in his symptoms his weight and height during these years. Primary investigations for celiac disease and wheat sensitivity were Negative. A gluten free diet was suggested for him with suspicious of non-celiac gluten sensitivity. After one-month significant improvement in gastrointestinal symptoms observed, there was also signs of catch-up growth in his height and weight, from 130 cm and 26.7 Kg to 132cm and 30.3 Kg, respectively) were observed. 

**Case 3**

A 3.5-year-old girl was referred by her general practitioner with a history of constipation, abdominal pain, and normal growth indices. Following primary investigations and a consultation with a pediatric gastroenterologist, she was referred to the immunology-allergy clinic for evaluation of probable food allergy. Due to negative investigations for CD and wheat allergy, a GFD was suggested for her with the suspicion of NCGS. Significant improvement in her symptoms was noticed within 2 weeks of starting the GFD.

**Case 4**

An 8.5-year-old girl was referred by a pediatric endocrinologist with a history of abdominal pain, short stature, and low weight. In her first visit, the patient’s height for age was under 3 percentiles and weight for age was on 5 percentiles. Primary investigations for CD and skin prick test (SPT) for wheat allergy were negative. A GFD was suggested with suspicion of NCGS, and significant improvement in abdominal pain was seen within 1 month of starting the diet. After 6 months, the patient’s growth indices also improved by 4 cm in height and 2 Kg in weight. 

**Case 5**

A 7-year-old girl was under observation of a pediatric endocrinologist with a history of FTT and abdominal pain since she was2 years old. As there was no improvement even with growth hormone therapy, the patient was evaluated for food allergy. Primary investigations for CD and skin prick test for wheat allergy were negative. A GFD was suggested with the suspicion of NCGS. A significant improvement in the patient’s symptoms was noticed within 1 month of starting the diet, and her growth indices improved: height from 106 cm to 109 cm, and weight from 17.2 Kg to 19.6 Kg.

## Discussion

Gluten is a complex molecule present in several grains, such as wheat, rye, and barley. Glutenin polymers and gliadin monomers are the main components of gluten. Both glutenin and gliadin contain high percentages of prolines (20%) and glutamines (40%) (15).

Gluten is responsible for several conditions, such as celiac disease, wheat allergy, and non-celiac gluten sensitivity. Adherence to gluten-free foods is the only available remedy for patients with the mentioned conditions (16).

NGCS is a condition associated with a wide range of both gastrointestinal and extra-gastrointestinal symptoms, which improve after gluten removal and reappear after gluten ingestion. These symptoms may include bloating, abdominal discomfort and pain, altered bowel habits, flatulence, rash, fatigue, headaches, mental disturbances, irritability, depression, bone and joint pain, and attention deficit disorder. Most NCGS symptoms are like those of IBS, a common condition affecting the digestive system and causing symptoms like stomach cramps, bloating, diarrhea and constipation, and NCGS. To date, the effect of a GFD on these conditions has not been explored (17). Despite typical gastrointestinal symptoms, NCGS has less frequent extra-intestinal manifestations in contrast to other gluten intolerance disorders. According to previous studies, fatigue is the most common extra-intestinal manifestation (18). Based on a previous meta-analysis conducted by Molina-Infante et al., signs of malabsorption are seen in NCGS patients, including weight loss, anemia, low ferritin, and vitamin deficiencies (folate, vitamin B12, and vitamin D) (19).

High-quality genetic studies on the NCGS population have not yet been performed. To the best of our knowledge, there are no data suggesting that the condition follows the same HLA-DQ2/-DQ8 association as CD. However, some studies suggest that approximately 50% of patients with non-celiac gastroenteropathy have the HLA-DQ2/-DQ8 haplotype compared with 30% of the general population (20).

There are no serological tests or histological features to diagnose NCGS, and the clinical workup for diagnosis of NCGS usually focuses initially on the exclusion of CD and wheat allergy. Unlike CD, there is no elevation in tTG-Ig. Skin prick tests also have negative results in contrast with wheat allergy (21,22). 

According to this condition, diagnosis is based on clinical manifestation and patients’ responses to gluten challenges. Definitive diagnosis is made when patients’ symptoms are under good control before the initiation of a gluten challenge (23).

The exact pathophysiology is still unclear, but according to previous studies, NCGS is not an autoimmune nor allergic disease. It is caused by innate and/or adaptive immune system dysregulation (24, 25). Three components are responsible for this disease, i.e. gluten, FODMAPs, and amylase-trypsin inhibitors. (2)

Five cases with a final diagnosis of NCGS are presented herein. All patients had FTT. Patients who had this condition for a longer duration had lower weights and heights for their ages. FTT may have been generated because of gastrointestinal manifestations and subsequent malabsorption. Consequently, early diagnosis of NCGS may prevent FTT symptoms in patients. Moreover, a GFD may provoke catch-up growth in NCGS patients with FTT. Failure to thrive is a known complication of CD (26); however, the relationship between NCGS and FTT is currently unknown. Based on the cases presented herein, NCGS may cause FTT in children during their growth years. 

The main limitation of this study was the short follow-up periods in the reported cases. 

In conclusion, NCGS must be considered in patients with prolonged gastrointestinal discomfort, such as abdominal pain, nausea, vomiting, and diarrhea. FTT manifestations must also be evaluated in these patients. NCGS may be misdiagnosed as IBS and cause extreme FTT over the long-term; therefore, early diagnosis and treatment may prevent short stature.

## Conflict of interests

The authors declare that they have no conflict of interest.
